# From Snapshots to Flipbook—Resolving the Dynamics of Ribosome Biogenesis with Chemical Probes

**DOI:** 10.3390/ijms21082998

**Published:** 2020-04-23

**Authors:** Lisa Kofler, Michael Prattes, Helmut Bergler

**Affiliations:** Institute of Molecular Biosciences, University of Graz, Humboldtstraße 50/EG, 8010 Graz, Austria; lisa.kofler@uni-graz.at (L.K.); michael.prattes@uni-graz.at (M.P.)

**Keywords:** ribosome biogenesis, ribosome biogenesis inhibitors, regulation of ribosome assembly, ribosome trafficking, chemical probing, feedback regulation

## Abstract

The synthesis of ribosomes is one of the central and most resource demanding processes in each living cell. As ribosome biogenesis is tightly linked with the regulation of the cell cycle, perturbation of ribosome formation can trigger severe diseases, including cancer. Eukaryotic ribosome biogenesis starts in the nucleolus with pre-rRNA transcription and the initial assembly steps, continues in the nucleoplasm and is finished in the cytoplasm. From start to end, this process is highly dynamic and finished within few minutes. Despite the tremendous progress made during the last decade, the coordination of the individual maturation steps is hard to unravel by a conventional methodology. In recent years small molecular compounds were identified that specifically block either rDNA transcription or distinct steps within the maturation pathway. As these inhibitors diffuse into the cell rapidly and block their target proteins within seconds, they represent excellent tools to investigate ribosome biogenesis. Here we review how the inhibitors affect ribosome biogenesis and discuss how these effects can be interpreted by taking the complex self-regulatory mechanisms of the pathway into account. With this we want to highlight the potential of low molecular weight inhibitors to approach the dynamic nature of the ribosome biogenesis pathway.

## 1. Introduction

Ribosomes are huge nanomachines responsible for the translation of the genetic information from mRNA into the amino acid sequence of proteins and are crucial for all living organisms. These ribonucleoprotein complexes consist of two subunits (40S and 60S for eukaryotic cytosolic ribosomes) with the small subunit harboring the decoding center and the large subunit containing the peptidyltransferase activity. Each subunit comprises a ribosomal RNA (rRNA) core that is shaped and shielded by ribosomal proteins (r-proteins). In eukaryotes, assembly of these r-proteins with pre-rRNAs and their conversion to mature rRNA occurs through a sophisticated maturation pathway that originates in the nucleolus and is finalized in the cytoplasm. For recent reviews on ribosome biogenesis in yeast see [1,2], for a recent review on human ribosome biogenesis see [3]. 

Maintaining a sufficient population of functioning ribosomes belongs to one of the main activities in living cells demonstrated by the fact that up to 60% of cellular transcriptions are spent for rRNA synthesis [4]. Indeed, ribosome biogenesis requires the activity of all three RNA polymerases, with Pol I and Pol III mainly dedicated to pre-rRNA synthesis and additionally, up to 50% of Pol II activity required for producing mRNAs for r-proteins. Owing to these high energy and resource demands, ribosome formation is tightly regulated in accordance with cell growth and proliferation mediated by several conserved regulatory pathways, including that of the Target of Rapamycin Complex 1 (TORC1 [5,6,7], reviewed in [8,9]), MYC and p53 (recently reviewed in [10]). An impairment of this interwoven regulatory network can lead to cancer development (recently reviewed in [11,12]) and/or severe developmental and neurodegenerative diseases (reviewed in [3,13,14]). 

**Figure 1:**
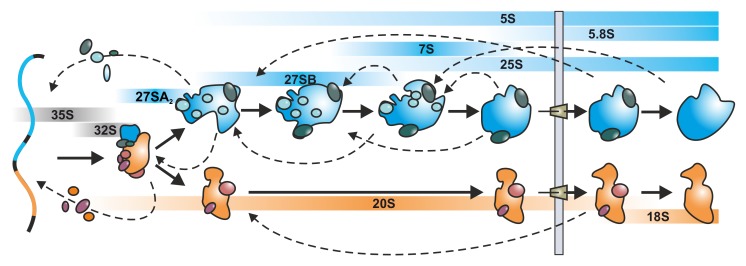
Schematic overview of eukaryotic ribosome biogenesis in yeast. The synthesis of ribosomes starts in the nucleolus with the transcription of the 35S pre-rRNA, containing the 18S, 5.8S and 25S rRNAs and co-transcriptional protein assembly to form the small subunit processome. Upon endonucleolytic cleavage within the 35S pre-rRNA, the maturation pathways of the small and the large subunit are separated. The pre-40S subunit (orange) is rapidly exported to the cytoplasm, while the pre-60S subunit (blue) has to undergo numerous rearrangements and processing steps before its nuclear export. In the cytoplasm the last maturation and quality control steps occur before the subunits become competent for joining and translation. The respective (pre-)rRNAs are depicted in blue (rRNAs of the 60S subunit) and orange bars (rRNAs of the 40S subunit), respectively. Dashed arrows symbolize the complex disassembly, recycling and association cycles of assembly factors during ribosome biogenesis.

Eukaryotic ribosome biogenesis requires the synthesis and assembly of four rRNAs (the 18S for the 40S and the 5S, 5.8S and 25S rRNA (yeast) or 28S rRNA (mammalian) for the 60S subunit) as well as the incorporation of 80 r-proteins. The incorporation of the individual components is orchestrated and coordinated by a plethora of proteins which act as assembly factors (also called auxiliary or maturation factors) including a large number of enzymes such as methyl-transferases, RNA helicases, ATPases and GTPases [15,16]. In addition, different snoRNPs are required for certain rRNA processing, folding and modification events (pseudouridinylation or methylation, reviewed in [16,17]). Since the vast majority of ribosomal assembly factors and r-proteins associate to the emerging subunits in the nucleus, they need to be efficiently imported from the cytoplasm which is facilitated by chaperones and importins (reviewed in [18]). Ribosome biogenesis thus is a highly complex pathway that has to coordinate the assembly and modification of numerous RNAs, transacting factors and r-proteins in three different cellular compartments (Figure 1). In this review, we highlight the potential of small molecular weight inhibitors to investigate the dynamic nature of this pathway. As ribosome biogenesis is best understood in the eukaryotic model organism *Saccharomyces cerevisiae*, we will focus mostly on data from yeast. Nevertheless, despite the additional levels of complexity, the herein described mechanisms are also at work in higher eukaryotes. In addition, we will only focus on inhibitors for which specificity for ribosome biogenesis was convincingly demonstrated.

### 1.1. A Short Overview of the rRNA Processing Cascade in Yeast 

**Figure 2:**
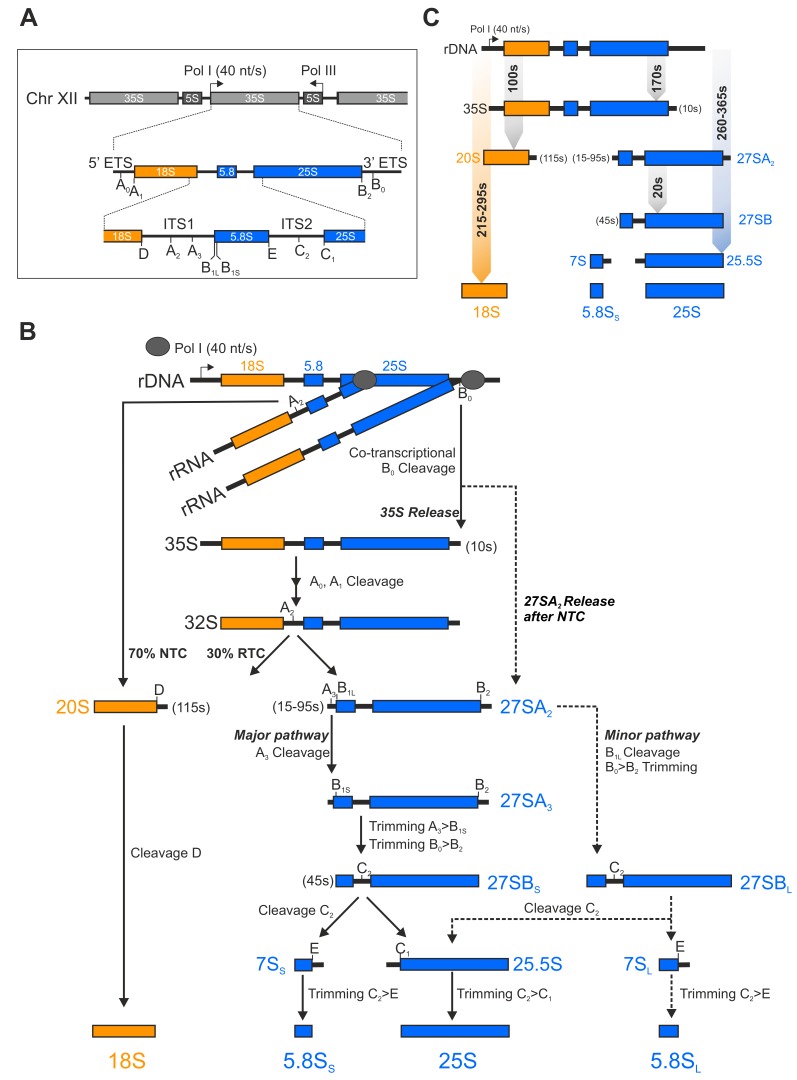
Eukaryotic rRNA processing in yeast. (**A**) Schematic depiction of the yeast rDNA locus on chromosome XII comprising 150 tandem repeats. Each repeat contains the sequence for the 5S rRNA which is transcribed by Pol III and the 35S pre-rRNA (comprising the 18S, 5.8S and 25S rRNA) which is synthesized by Pol I with a transcription rate of about 40 nucleotides per second in the opposite direction [20]. (**B**) Schematic overview of the 35S pre-rRNA processing cascade. During the exponential growth phase 70% of the nascent transcripts are co-transcriptionally cleaved at the A_2_ site (NTC: nascent transcript cleavage), while 30% are cleaved post-transcriptionally (RTC: released transcript cleavage) [20]. (**C**) Lifetimes (brackets) and processing times of pre-rRNAs in seconds (s). Koš and Tollervey (2010) determined the lifetimes and processing times of pre-RNAs via metabolic labeling [20]. The processing time of the 35S pre-rRNA to the indicated rRNAs is given in a range, since it is dependent on whether the A_2_ cleavage occurs co- or post-transcriptionally.

The starting point of ribosome assembly is the nucleolus, a membrane-less sub-compartment of the nucleus where the rDNA transcription and the first protein assembly events take place (Figure 1). While the 5S rRNA of the large subunit is transcribed by Pol III and incorporated as a nucleoprotein particle with the r-proteins uL18 and uL5 (nomenclature [19]), the major transcriptional activity is provided by Pol I which transcribes a long 35S polycistronic precursor RNA containing the sequence for the mature 18S, 5.8S and 25S rRNA, flanked and separated by external (5’ and 3’ETS) and internal transcribed spacers (ITS1 and 2), respectively (Figure 2A). 

The first pre-ribosome maturation factors and r-proteins already bind co-transcriptionally to the nascent 35S pre-rRNA and form the earliest pre-ribosomal particle called small subunit (SSU)-processome in which the initial rRNA modification and processing steps take place [21,22,23,24,25,26]. A specific endonucleolytic cleavage at the A_2_ site within the ITS1 separates the 18S precursor (called 20S pre-rRNA) from the 27S pre-rRNA which represents the common precursor of the 5.8S and the 25S rRNA. This processing event thus separates the biogenesis pathways of the small 40S from the large 60S subunit. The A_2_ cleavage can occur both, co- and post-transcriptionally (Figure 2B) [20,27,28]. Koš and Tollervey (2010) could demonstrate that during exponential growth up to 70% of the nascent transcripts are cleaved co-transcriptionally [20]. After A_2_ processing, the pre-40S particle is released and rapidly exported into the cytoplasm, where the 20S pre-rRNA is cleaved at the D site to generate the mature 18S rRNA. In contrast, the pre-60S particle faces a complex series of rRNA processing steps on its journey from the nucleolus to the nucleoplasm and has to undergo massive restructuring before it can be exported to the cytoplasm. The rRNA processing reactions involve: (1) endonucleolytic cleavage of 27SA_2_ at site A_3_ and removal of the 3’ETS to generate 27SA_3_ pre-rRNA, (2) exonucleolytic trimming of the remaining ITS1 yielding 27SB pre-rRNA, (3) cleavage at site C_2_ resulting in the 7S and 25.5S pre-rRNAs and (4) exonucleolytic removal of ITS2 which gives rise to the mature 25S and the 6S pre-rRNA, which is finally trimmed in the cytoplasm to the mature 5.8S rRNA (Figure 2B; ITS2 processing was recently reviewed in [29]). Although the processing cascade has a canonical order, two alternative pathways exist to process the 27SA_2_ pre-rRNA. The vast majority of 27SA_2_ molecules are cleaved at the A_3_ site and processed as mentioned previously, yielding 27SB_S_, while a small fraction of 27SA_2_ is directly cleaved at B_1L_ which generates a 5’ extended version of 27SB (27SB_L_). Subsequently, both 27SB variants are cleaved at C_2_ and follow the same processing pathway to the mature 5.8S_(S/L)_ and 25S rRNAs (Figure 2B, for a detailed review on the rRNA processing cascade see [30,31]). Only those particles, which passed all nuclear maturation stages gain export competence and are, hence, efficiently transported through the nuclear pore complexes by dedicated export factors (recently reviewed in [32]). In the cytoplasm the remaining assembly factors are released and eventually replaced by the last r-proteins before the subunits can join to fulfill their role in translation [33,34,35,36,37,38,39,40,41,42].

### 1.2. 250 Assembly Factors Empower Pre-Ribosomal Particle Maturation

To guarantee efficient and error-free formation of translation competent ribosomes, the pathway is organized in a hierarchical manner with a strict order of assembly and disassembly of the maturation factors (e.g., [43,44], recently reviewed in [2]). This means that early factors have to leave the particle at the right stage to make the binding sites accessible for later joining factors. Since this mechanism establishes a strict temporal and spatial order of events, it allows proofreading of previous maturation steps and builds checkpoints before irreversible reactions take place. An example and probably the most important checkpoint is the nuclear export of pre-ribosomal particles. Particles that have not successfully accomplished all nuclear maturation steps, can normally not be exported through the nuclear pore complexes and are degraded [45], although certain exceptions to this rule were recently described [46,47,48]. Thus, the separation of nuclear and cytoplasmic steps of ribosome biogenesis prevents the participation of premature particles in translation but demands tight coordination and communication between the two compartments. 

Most of our current knowledge about the hierarchical order of maturation factors binding and dissociating within the pathway was obtained by tandem affinity purification using bait proteins from different maturation stages combined with mass spectrometry and pre-rRNA processing studies. These analyses permitted identification of new assembly factors and their assignment to a specific step in the ribosome biogenesis cascade, according to the co-enriched rRNA intermediates that served as “landmarks” (e.g., [49,50]; reviewed in [1]). Furthermore, tandem affinity purification from genetically manipulated cells demonstrated the interdependency among the assembly factors and their hierarchical recruitment to the pre-ribosomes [49,51,52,53,54,55]. During the maturation process the particles undergo massive structural changes as demonstrated by groundbreaking structural analysis of different pre-ribosomal particles by cryo-EM [22,24,25,26,39,56,57,58,59,60,61,62]. This whole maturation cascade is driven by about 250 different assembly factors each required at a distinct stage. As most of these factors are essential, the ribosome biogenesis pathway represents a vast pool of potential targets for chemical inhibition.

### 1.3. Ribosome Biogenesis Inhibitors—Chemical Probes to Unravel Ribosome Biogenesis

Up to now many assembly factors can only be assigned to distinct maturation steps in groups and full resolution of the binding hierarchy has still to be achieved. This is due to the high rate at which the individual reactions take place. A single yeast cell, for example, has to synthesize more than 2000 ribosomes each minute to cope with the high demands during exponential growth [4]. Thus, ribosome biogenesis is a very dynamic process with the formation of the 25S rRNA from the 35S pre-rRNA taking only about six minutes (Figure 2C, [20]). Consequently, it is challenging to monitor the dynamic changes during ribosome assembly by classical genetics and biochemical means like purification of precursor particles from genetically manipulated cells, because the onset of genetic perturbation is too slow. Furthermore, the high interdependency among the assembly factors in combination with the fast execution of the individual steps makes the identification of primary effects challenging. The accurate assignment of observed perturbations to individual maturation steps therefore requires fast inactivation of a specific target and the immediate monitoring of the induced changes over a period of time. Due to their fast diffusion into the cell and specific binding to their target, small molecule inhibitors perturb ribosome biogenesis within seconds and thus enable its investigation with high temporal resolution. By hindering supply with ribosomal proteins, any translational inhibitor will also lead to the inhibition of ribosome biogenesis (as for example rRNA processing defects are reported as a result of cycloheximide treatment [63,64]). In contrast, specific ribosome biogenesis inhibitors will block ribosome maturation directly and will therefore not affect translation after short term treatment or in an in vitro translation system. As ribosomes are relatively stable, ribosome biogenesis inhibitors will not harm resting cells, but preferentially target fast proliferating cells. Thus, ribosome biogenesis represents an Achilles heel of tumor cells that makes them sensitive to inhibition of this pathway.

Several inhibitors of the very first step of ribosome biogenesis, the transcription of rDNA, were described in recent years. These compounds include BMH-21, CX-3543 and CX5461 which show different mechanisms for inhibiting Polymerase I transcription. BMH-21 for example, intercalates into GC rich DNA [65]. CX-3543, also known as quarfloxin, inhibits transcription elongation by blocking nucleolin 1 interaction with rDNA [66]. The compound CX-5461 was shown to prevent recruitment of the transcription factor SL1 to the promotors of rDNA [67]. Both, CX-5461 and CX-3543 seem to bind to and stabilize G-quadruplex DNA structures and lead to induction of the DNA damage response [68]. Strikingly, CX-5461 exhibits preferential toxicity to tumor cells compared to primary cells and induced apoptosis and senescence through p53-dependent and -independent mechanisms [69]. Thus, Polymerase I inhibitors are very promising candidates for tumor therapy and affect the growth of cancer cells not responding to other treatments [64,65]. Without doubt, such inhibitors will represent a major step forward in the clinical treatment of malignancies. For detailed reviews on Polymerase I inhibitors see [69,70].

The large number of ribosomal maturation factors provides a rich, but mostly unexplored repertoire to identify novel chemotherapeutic drugs. Besides being candidates as future anti-cancer drugs, inhibitors that block the pathway at a later stage than transcription bear the potential to deliver detailed insights into the dynamics and order of the individual steps of the ribosomal maturation pathway. 

The first small molecular weight compound identified to target eukaryotic ribosome maturation was diazaborine [71]. Diazaborine specifically inhibits the formation of the 60S subunit by targeting the cytoplasmic AAA-ATPase Drg1 which consists of an N-domain and two ATPase domains [72,73,74]. Diazaborine blocks ATP hydrolysis in the second ATPase domain of Drg1, which is essential for the release of the assembly factor Rlp24 from pre-60S particles shortly after nuclear export [41,75,76]. As this release reaction is the prerequisite for all downstream events of the cytoplasmic 60S maturation cascade, diazaborine not only prevents the release and recycling of Rlp24, but also of all other assembly factors that accompany the pre-60S particle into the cytoplasm. These shuttling factors become trapped on the exported particle and are therefore not capable to reenter the nucleus which leads to a secondary block at nucleolar maturation stages. Considering the complex effects of diazaborine on particles from early nucleolar to late cytoplasmic stages in a chronological order provided deep insights into the dynamics and kinetics of ribosome biogenesis and will be reviewed in detail below. 

Another recently identified class of eukaryotic ribosome biogenesis inhibitors are the ribozinoindoles (short Rbins) which target the dynein-like AAA-ATPase Mdn1 in *Schizosaccharomyces pombe* [77], the ortholog of Rea1 from *Saccharomyces cerevisiae* [78,79]. Rea1 seems to be a multifunctional ATPase involved in the release of the nucleoplasmic factor Rsa4 as well as the nucleolar factor Ytm1, possibly in conjunction with Erb1 [78,79,80]. The inhibition of Mdn1 by Rbin-1 paves the way for detailed studies on the exact function of Rea1/Mdn1 and bears the potential to dissect its complex roles in nucleolar and nucleoplasmic maturations stages [77]. The detailed effects of Rbins on pre-ribosomal particle maturation and rRNA processing in fission yeast are described below.

Very recently about 100 novel inhibitors of eukaryotic ribosome formation were reported that act at different stages within the pathway [81]. Although the exact targets of these compounds are still elusive, they provide a promising toolbox to examine the eukaryotic ribosome biogenesis pathway step by step. In the next section we highlight the benefits of low molecular weight inhibitors to study the complex pathway of ribosome biogenesis and how they can be used to gain deeper insights into the order and dynamics of the assembly process. 

## 2. A Practical Guide to Read the Effects of Inhibitors on Ribosome Biogenesis 

**Figure 3:**
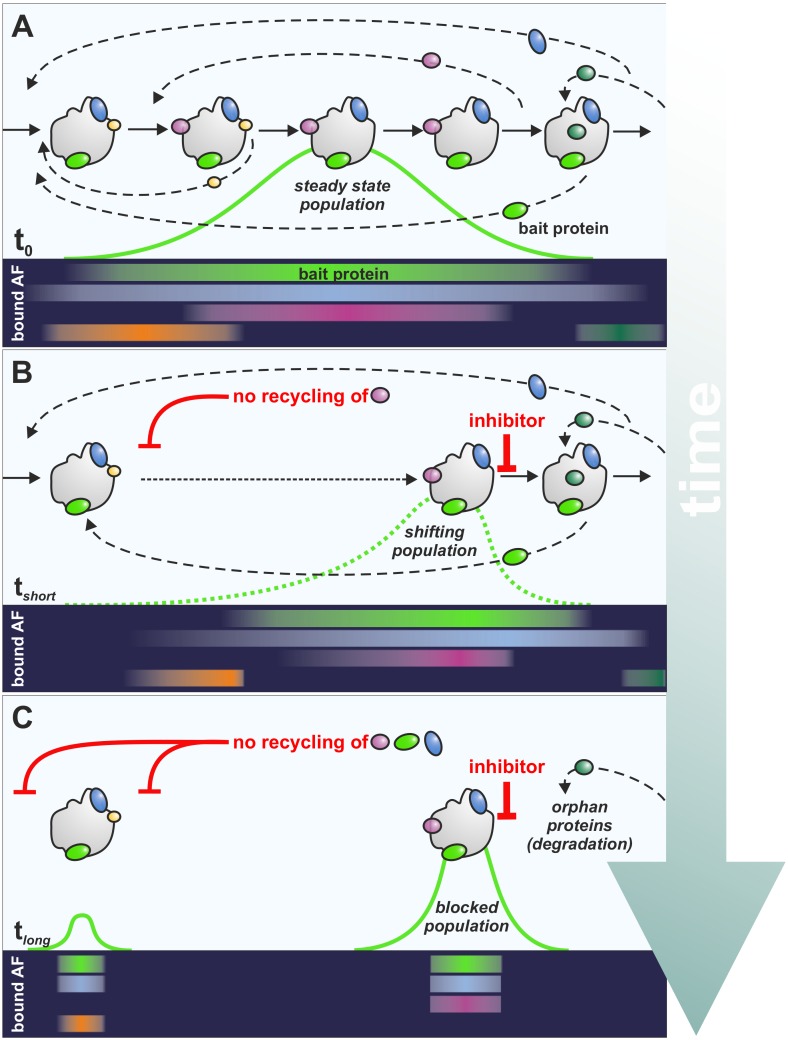
Impact of inhibitor treatment on eukaryotic ribosome biogenesis highlights the dynamics of the pathway. (**A**) The purification of the bait protein (light green protein) results in the copurification of a steady state particle population (indicated by curves) containing several distinct particles representing different maturation stages. (**B**) Upon short term inhibitor treatment (t_short_) particles accumulate at the stage where the protein target is implicated in ribosome biogenesis, resulting in an entrapment of assembly factors that would be released after the block (magenta protein). This entrapment can cause a secondary blockage at earlier stages. Since downstream processes are not hindered at the time of drug application, these maturation steps still proceed for a certain period of time. Hence, assembly factors that leave the particle before the block (orange protein) will decrease in the particle population. However, also assembly factors that would bind downstream of the block (dark green protein) will decrease in the purification. Assembly factors that bind before the bait protein and would be released after the removal of the bait protein (blue protein) remain unchanged. (**C**) After long term treatment (t_long_) a segregation of the particle population occurs, resulting in early population lacking factors (magenta factor) that are entrapped on the later population. Unassembled (orphan) proteins which cannot find their designated particle anymore (dark green factor) become degraded or precipitate. Dashed arrows symbolize the complex disassembly, recycling and association cycles of assembly factors during ribosome biogenesis. Bars represent particle-bound state of assembly factors (bound AF).

Pre-ribosomal particles affinity-purified via an assembly factor as bait protein (Figure 3, light green factor) do not represent a homogenous particle population with a distinct protein and RNA content but consist of several distinct ribosomal precursor particles representing different maturation stages. These particles differ in their composition depending on the stage the bait protein joins and leaves the pathway. Hence, the longer the assembly factor used as bait protein is associated with the pre-ribosome, the more heterogeneous the steady state population of the purified particles. The co-purified particles thus represent all the different maturation events that occur between the binding and the release of the respective assembly factor (Figure 3A). For simplicity, one could assume a Gaussian distribution for most of the particles when they transit trough the maturation cascade. However, this will not hold true when the bait protein passes through a rate limiting step of the pathway, which will lead to an accumulation of particles upstream of the bottle neck. In contrast, particles after a rate limiting step of maturation will only be found in low abundancy and are, thus, underrepresented. Similarly, inhibitors prevent progression of the particles to more mature forms and therefore also result in an accumulation of particles immediately upstream of the blockage while downstream particles are reduced. 

Owing to the inhibitor induced block in the maturation cascade, factors get trapped on the particle not capable of recycling (Figure 3B). This leads to their depletion at the step where they usually reenter the maturation path, which—if these proteins are essential—will result in a secondary block upstream of the inhibitor mediated blockage. Therefore, blocking ribosome biogenesis always results in an additional inhibition of maturation steps upstream of the block. The impact of the recycling defect on earlier steps depends on the relative distance between the disassembly of the respective factor and the inhibited step. Thus, the factor which should be the first leaving the particle after the block (Figure 3B, magenta factor), results in the fastest (strongest) depletion and, thus, leads to the first effect on earlier particles. 

This mechanism is likely to be valid also for other perturbations of the pathway, such as depletion of maturation factors or during regulatory processes when, for example, nutrients become limited and TOR activity ceases [82]. Since the amount of free assembly factors not bound to precursor particles is generally very low, the onset of secondary effects occurs dramatically fast. This “rebound effect” [63] is a fundamental regulatory property of the ribosome biogenesis pathway allowing rapid response and coordination of early and late stages when problems arise from shortage of ribosomal proteins or maturation factors. However, this feedback mechanism is not only limited to secondary effects but will be transmitted further up step by step to the very first pre-ribosomal particles. As a consequence, also co-transcriptional cleavage of the nascent transcript can be inhibited resulting in an increase of 35S pre-rRNA. This mechanism might be the cause of the often-observed accumulation of 35S pre-rRNA in mutants of the 60S biogenesis pathway. 

Particles downstream of the inhibitor initiated blockage at the time of drug application transit unimpeded through the maturation path and assembly factors are recycled as expected (Figure 3B). This leads to a shrinkage of the particle population downstream of the inhibitor induced blockage. Concomitantly, particles downstream of the secondary block can mature unhindered until they hit the block induced by the inhibitor, where they accumulate. As a consequence, a segregation of the particle population over time occurs, resulting in an early population lacking factors that are trapped on the later, inhibitor-stalled population (Figure 3C). Thus, the blockage leads to the accumulation of specific particle populations with a distinct protein and RNA composition. As the pre-rRNAs present in the particles between the primary and secondary blockage are further processed while those pre-rRNAs that are present in the inhibited particle population are enriched, the fastest accumulating rRNA-precursor can be used as a “landmark” to provide information about the maturation step that is blocked by the inhibitor. 

Due to the rapid transition of pre-ribosomal particles through the maturation cascade, assembly factors show highly dynamic association and dissociation, with the mean residence time depending on the exact stage of association and dissociation but clearly in the seconds to minutes range. The accumulation of distinct proteins on purified precursor particles indicates that these proteins are part of the accumulated particle population (Figure 3, magenta factor). In contrast, decrease on ribosomal intermediates can either signal that the factor assembles prior to the bait protein and leaves the particle upstream of the block (Figure 3, orange factor) or that the respective protein joins the maturation downstream of the inhibited stage (Figure 3, dark green factor). In both cases there is a decreased population that contains the respective assembly factor. Assembly factors that bind prior to the bait protein and disassemble after the bait protein are unchanged (Figure 3, blue factor). The extent to which the level of a factor changes in a shifting population indicates the distance of the assembly or disassembly event relative to the inhibitor-mediated block. Factors that bind shortly prior to the block will show a stronger relative accumulation than factors that where already assembled further upstream. On the contrary, factors that should leave the particle shortly after the block would decrease to a greater extent than ones that disassemble later. Thus, determining the relative protein accumulation can unravel the hierarchical order of assembly factors. If such analyses are performed with overlapping bait proteins and/or in sharp time series, very detailed information about association and dissociation characteristics of many factors can be obtained. In addition, factors can be assigned to specific reactions in the pathway, based on similar binding or dissociation kinetics [63]. 

Hence, the interpretation of the compositional changes of a shifting particle population requires recycling events to be taken into account and highlights the great potential of small molecular weight inhibitors. The fast onset of inhibition upon treatment with those inhibitors allows, for the first time, the dissection of the individual phases of this rebounding cascade step by step, which is not possible by conventional biochemical or genetic means and provides additional insights into (up to now unknown) checkpoints in ribosome maturation.

## 3. Examples for Ribosome Probing

In the following section we exemplify the above-mentioned effects of ribosome biogenesis inhibitors. We will focus on the best studied inhibitors diazaborine and Rbin-1, both targeting eukaryotic large subunit biogenesis, and will discuss how they contribute to a better understanding of the impact of perturbations within the pathway and to unravel the dynamics of the ribosome assembly pathway. 

### 3.1. Rbin-1 Blocks Rsa4 Release by Mdn1 (Rea1) in the Nucleoplasm

The AAA-ATPase Mdn1 from *Schizosaccharomyces pombe* is, like its *Saccharomyces cerevisiae* ortholog Rea1, responsible for the release of the WD repeat protein Rsa4 from late nucleoplasmic pre-60S particles [77,78,79,83]. This reaction is required for Rsa4 recycling for subsequent rounds of ribosome biogenesis (recently reviewed in [84]). Rea1 is crucial for a major structural transition of the pre-60S particle in the nucleoplasm, leading to a 180° rotation of the 5S RNP to its mature position [61,62]. In addition, Rea1 is also implicated in the release of the WD repeat protein Ytm1, in a reaction which was proposed to be coupled to the extraction of Erb1 [80,85]. Erb1 protrudes deeply into the 60S precursor particle and serves as a hub protein for many different maturation factors. Conceivably, the removal of Erb1 also initiates the release of several associated proteins before the particle transits from the nucleolus to the nucleoplasm. The coupled release of Ytm1 and Erb1 could provide an explanation why Rea1 requires such a long lever to generate enough mechanical force [60,80].

The use of Rbin-1 and derivatives thereof gave structural and mechanistic insights into the catalytical cycle of Mdn1 [77,83] and uncovered its function in ribosome biogenesis in fission yeast (recently reviewed in [84,86]). Inhibition of Mdn1 by Rbin-1 resulted in an accumulation of 27S and 7S pre-rRNAs, which goes in line with a blocked transition of the nucleolar particle to the nucleoplasm upon treatment with Rbin-1 [77]. Similar to the observation for Rea1 [79,80], the substrate-binding MIDAS domain of Mdn1 interacts with the MIDO-domain of Rsa4 and, albeit in a weaker manner, with that of Ytm1 [77], indicating a conserved mechanism of release for both WD repeat proteins. Short-term treatment with Rbin-1 resulted in a transient accumulation of Rsa4 on Rix1 containing particles after 15 minutes, while Ytm1 remained constant. This was followed by a decrease of both assembly factors after long-term treatment, which also occurred in a *mdn1-ts* mutant strain at the restrictive temperature [77]. Furthermore, examination of the Rbin-1 effects on the earlier, nucleolar Nsa1-particle revealed decreased levels for Rix7 and Ppp1 (the orthologue of Nop7 in *S. cerevisiae*), but no alterations of Ytm1 levels upon Rbin-1 treatment. In addition, the treatment of fission yeast with Rbin-1 also resulted in a redistribution of Rix7 and Ppp1 from the nucleolus to the nucleoplasm [77]. In contrast, Ytm1 and Nsa1 localization was not affected. These findings led to the hypothesis that Mdn1 is required for the recruitment of assembly factors to nucleolar pre-60S particles prior to the removal of Ytm1. Inhibition of this step would then lead to a secondary effect at later nucleoplasmic stages explaining the decrease of Rsa4 and Ytm1 upon long term Rbin-1 treatment [77]. Considering the strong rebound effects occurring within the ribosome biogenesis pathway, the effects on Nsa1 particles could also be a consequence of a Rsa4 recycling defect from the nucleoplasm, which will result in a secondary, nucleolar block. This secondary block will prevent particles from entering downstream maturation and hence result in the observed decrease of Rsa4 and Ytm1 on nucleoplasmic Rix1 particles. To test this hypothesis shorter treatment periods and kinetic analyses of the drug effects would be necessary. 

### 3.2. Dissecting the Complex Interwoven Pathway by the Help of Diazaborine 

The best investigated ribosome biogenesis inhibitor to date is diazaborine. The effects and dynamics of inhibition by diazaborine were investigated from early nucleolar and late cytoplasmic stages using Northern blotting, cell biological and proteomic tools [63,71,72,73]. The finding that diazaborine treatment blocks recycling of shuttling proteins provides the unique opportunity to gain detailed insights into the coordination between cytoplasmic and nucle(ol)ar steps of ribosome biogenesis. Moreover, the impact of shuttling protein depletion on nucleolar maturation was investigated for early and late nucleolar particles using Noc2 and Nsa1 as bait proteins. This analysis provided insights into the type of particles accumulating upon shuttling protein depletion and how this depletion is transmitted to even earlier stages [63]. 

#### 3.2.1. Tracing Pre-60S Particle Maturation from the Nucleolus to the Cytoplasm

By inhibiting the ATPase activity of Drg1, diazaborine blocks the cytoplasmic release of Rlp24 which is the obligatory initial step of cytoplasmic maturation. As a consequence, shuttling factors cannot be recycled which prevents freshly made early nucleolar particles from entering the maturation path. Therefore, only particles that have already assembled all essential shuttling proteins at the time of drug application can undergo maturation until they hit the step where Rlp24 should be released [63,73,75,76]. This allows following the maturation pathway by purifying pre-60S particles with a TAP tag on a suitable shuttling protein after different periods of drug treatment. The range of maturation steps that can be monitored by this approach strongly depends on the chosen maturation factor. 

The shuttling GTPase Nog1 proved to be highly suitable for this task. Nog1 joins the maturation cascade at the nucleolar stage, inserts its C-terminal tail into the polypeptide exit tunnel and accompanies the particle until its export into the cytoplasm [58,87]. In the cytoplasm, Nog1 is released from the particle shortly after Rlp24 in a reaction that requires GTP hydrolysis [42]. Given the fact that Nog1 is among the factors that get trapped on the exported cytoplasmic particle upon inhibition of Drg1, it is an ideal candidate to follow the maturation pathway of the pre-60S particle from the nucleolar to the first cytoplasmic step after diazaborine treatment. Interestingly, Nog1 is retained in the nucleolus after TOR1 inactivation and nutrient depletion, suggesting that it is under the control of the TORC1 pathway and has a crucial regulatory role in ribosome assembly [7,82,88].

**Figure 4:**
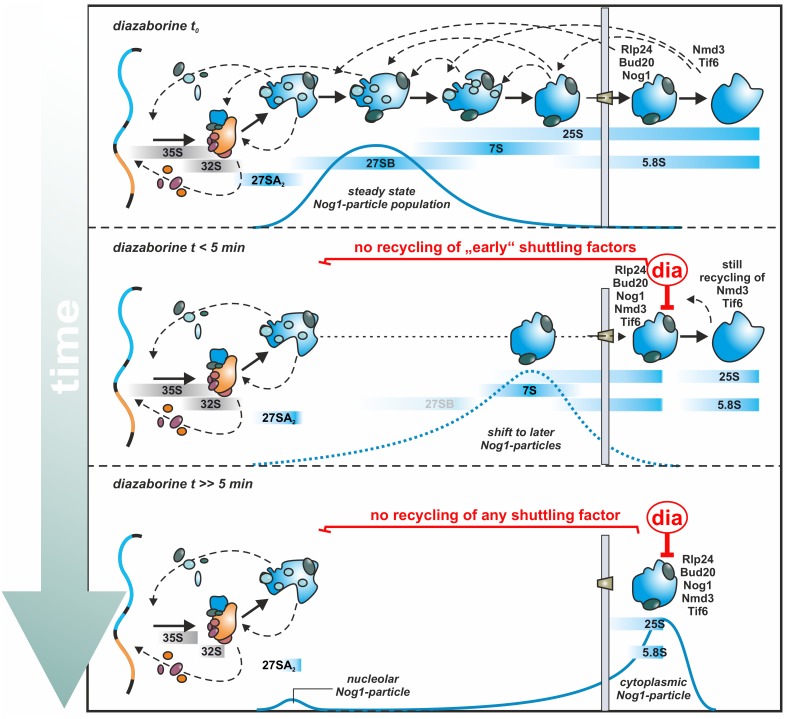
Diazaborine treatment allows tracing the ribosome biogenesis pathway from the nucleolar to the early cytoplasmic stage. While the steady state population of Nog1 in untreated cells (diazaborine t_0_) mainly contains 27SB pre-rRNA representing late nucleolar and early nucleoplasmic particles, treatment with diazaborine leads to a steady shift to a cytoplasmic population shortly after export. Since Nmd3 and Tif6 are the last two factors that leave the 60S-precursor before subunit joining, their cytoplasmic pool is significantly larger than the ones of Rlp24, Bud20 and Nog1, which dissociate shortly after export. As a consequence, significant amounts of Nmd3 and Tif6 can still be recycled shortly after drug application, while the recycling of Rlp24, Bud20 and Nog1 is blocked immediately (diazaborine t < 5 min). This fact allows tracking the particles that already contained Nog1 at the time of diazaborine application over time. The small nucleolar peak of Nog1 originates from de novo synthesis and import of Nog1, which is not affected by diazaborine and becomes detectable after about 15 minutes of drug treatment (diazaborine t >>5 min) [63].

The isolation of the Nog1-particle from untreated cells results in the enrichment of its steady state population comprising mostly the late nucleolar stage (Figure 4, diazaborine t_0_). This is obvious from published data which show a strong association with late nucleolar and early nucleoplasmic factors (Nsa1, Noc3, Nop7, Nsa2 and Nog2) but a weak association with very early nucleolar factors like Noc1 and Noc2 and the absence of export factors. This goes in line with the presence of very little 27SA_2_ pre-rRNA, plenty of 27SB pre-rRNA and also some 25S rRNA and is consistent with a steady state localization of Nog1 in the nucleolus and nucleoplasm [49,63,87,89,90]. 

Upon drug treatment, nucleolar supply of Nog1 ceases which is obvious by its depletion from early particles (demonstrated with Noc2-TAP and Nsa1-TAP) [63]. Consequently, only particles that already contain Nog1 at the moment of inhibition are able to pass through the maturation pathway until they reach the diazaborine-mediated block in the cytoplasm (Figure 4, diazaborine t < 5 min). This allowed following the maturation of the large subunit by purification of Nog1-containing particles after different treatment periods. The transition is manifested by the steady decrease of nucleolar factors (e.g., Noc1, Noc2, Nop7) and increase of the export factors (e.g., Mex67, Nmd3). In line with these results, 27SA_2_, 27SB and 7S pre- rRNAs decrease over time while the 25S and 5.8S rRNA signals increase (Figure 4) [63]. The mostly nucleoplasmic proteins Nsa2, and Nog2 reached the highest levels after about five minutes of diazaborine treatment, when 27S already starts to decline. This suggests that cleavage at ITS2 and binding of these factors is temporally tightly linked. Rsa4 is the last nucleoplasmic factor to join and associates with the particle at a stage when 7S pre-rRNA already starts to decline. The export adapter Nmd3 constantly increased over the treatment period, demonstrating that an increasing portion of the Nog1-particle gains export competence [63]. 

This kinetic analysis of the transition of Nog1 through the maturation cascade provides a detailed picture of the order of assembly and disassembly events and allowed setting it into the context of pre-rRNA processing. The extent at which the maturation factors accumulate or decrease indicate their hierarchical order of joining and release. Intriguingly, assembly factors participating in the same maturation step show similar kinetics [63]. This observation also allows assignment of maturation factors with unknown functions to specific reactions. Only after 15 minutes of incubation with the drug de novo synthesized Nog1 was observed, which associated with 27SA_2_ pre-rRNA (Figure 4, diazaborine t >> 5 min) [63]. This finding suggests that the prevalent assembly activity is driven by recycled and not freshly synthesized maturation factors. 

#### 3.2.2. Coordination of Particle Export and Downstream Maturation in the Cytoplasm

As mentioned above, Nog1 is released in the cytoplasm shortly after Rlp24 [42]. Consequently, only a very minor Nog1-containing particle population lacking Rlp24 has to exist which could undergo maturation after diazaborine treatment and liberate Nog1 for recycling into the nucleus. In contrast, shuttling proteins that are normally released later in the cytoplasm are present on a larger pool of particles that can mature unhindered in the presence of diazaborine. Therefore, a larger pool of these shuttling proteins exists, which can still be recycled in the presence of the inhibitor. This is especially true for Nmd3 and Tif6 being the last proteins to be released from the pre-ribosomal particle [36,39,40]. Therefore, the cytoplasmic pool of the later released shuttling factors is much larger than that of early released shuttling factors and the onset of their nuclear depletion is slower than that of Nog1 (Figure 4). 

Nmd3 provides the nuclear export signal of the pre-60S particle for recognition by the exportin XpoI/Crm1 and is likely the last factor that associates with the particle prior to export. Since Nmd3 is released only shortly before Tif6, which is the last assembly factor that dissociates from the maturing particle, the majority of Nmd3 is present in the cytoplasm as obvious by its steady state localization [39,91]. Thus, at the time of diazaborine application, most of the Nmd3 molecules are bound to cytoplasmic particles already beyond the drug sensitive step and can be recycled unhindered. This allows the export of nuclear particles to continue for a prolonged period of time. However, once this pool is exhausted, export will cease and pre-60S particles will also accumulate prior to export. This is consistent with microscopic studies showing that a small portion of Nog1-GFP and Bud20-GFP are retained in the nucleus even after prolonged treatment with diazaborine [63]. Moreover, structural studies showed that overexpression of truncated Rlp24, which prevents Drg1 binding to pre-60S particles, leads to early cytoplasmic pre-60S particles containing Nmd3, but also to a smaller portion of late nuclear pre-60S particles that lack Nmd3 but contain Nog2 and Nsa2 proteins [56]. An identical set of particles (e.g., a larger portion of early cytoplasmic and a smaller portion of late nuclear particles containing Nog2 and Nsa2) were also isolated after diazaborine treatment (Bergler and Warren labs, unpublished results). As the replacement of the nucleoplasmic GTPase Nog2 by Nmd3 [92] and subsequent binding of the exportin XpoI/Crm1 is thought to represent the last nuclear step of pre-60S maturation [93,94,95], the presence of Nog2 and Nsa2 on these particles suggests that Nmd3 and/or possibly other export factors are not only required for XpoI/Crm1 dependent export itself, but also for the release and recycling of late nucleoplasmic factors. The nuclear depletion of Nmd3 therefore likely represents a communication mechanism between the cytoplasm and the nucleus and provides a feedback mechanism, which hinders the export of further particles if cytoplasmic maturation steps cannot be accomplished.

## 4. Cellular Response to Inhibition of Ribosome Biogenesis

As outlined above, perturbations in the ribosome biogenesis pathway caused by chemical inhibition will result in accumulation of distinct pre-ribosomal particle populations, while downstream intermediates are largely depleted. This will lead to accumulation of unassembled (orphan) r-proteins or assembly factors and eventually to stalled pre-ribosomal particles. Therefore, cells have developed mechanisms to prevent protein toxicity and to avoid extensive accumulation of stalled pre-ribosomal particles. These surveillance systems are briefly described in the next sections.

### 4.1. Orphan Proteins Signal Stress Owing to Blocked Ribosome Biogenesis

Due to its fast progression and the high expression levels of r-proteins, ribosome biogenesis is highly prone to cause an imbalance in protein homeostasis. A block in ribosome biogenesis causes a fast decrease of downstream precursor particles and r-proteins and assembly factors cannot be incorporated. This results in their accumulation in free form and leads to their aggregation and eventual degradation [96,97]. Accordingly, inhibition by small molecule inhibitors results in an increase of unassembled r-proteins and/or assembly factors because their target particle population shrinks. After diazaborine treatment Rix1 and Rsa4 accumulate in free form ([63]; our unpublished results) and other maturation factors (e.g., Nsa2, Nog2, Nop53 and Sda1) precipitate [96] as a consequence of the secondary block in the nucleolus, while eL24 and eL42 precipitate as a consequence of the primary block shortly after export [96]. 

To prevent proteotoxicity, cells have evolved control mechanisms to precisely sense an excess of unassembled r-proteins. Recently, Hsf1, a key regulator of proteostasis [98] was shown to specifically activate a stress pathway that downregulates r-protein genes and concomitantly upregulates a Hsf1 specific regulon including chaperones, nuclear/cytoplasmic aggregases and components of the proteasome in response to aggregated r-proteins [99,100,101]. This stress response pathway is dependent on de novo synthesis of r-proteins that accumulate in insoluble fractions where also Ifh1, a transcription activator of r-proteins, aggregates and therefore is sequestered from r-protein promoter. Strikingly, this stress response is very rapid but transient, which is shown by the fact that upregulation of the Hsf1 regulon and downregulation of r-protein transcription occurs within a few minutes, but is reverted to normal levels after 20 minutes [64]. This reveals that cells constantly monitor their unassembled r-protein pool to ensure ongoing ribosome biogenesis, but concomitantly prevent proteotoxicity. 

### 4.2. Degradation of Aberrant Ribosomal Precursors

Another control system that monitors ribosome assembly is the RNA surveillance system that degrades aberrant RNA molecules or RNA-protein complexes. One central component of this RNA surveillance machinery is the exosome, which is present in both, the nucleus and the cytoplasm (reviewed in [102]). Strikingly, this complex is not only implicated in the 3’ – 5’ degradation of several different RNA species but is also required for the precise 3’trimming of precursor RNAs to generate their mature 3’ends. One example is the processing of the 7S pre-rRNA, where the exosome removes the remaining sequence of the ITS2 to produce the 3’end of the 5.8S rRNA [103,104,105,106] (Figure 2B, recently reviewed in [29]). To facilitate RNA degradation the TRAMP complex attaches a short 3’poly(A)tail that enhances the processivity of the exosome [107,108] (reviewed in [102,109]). It was suggested that the kinetics of ribosome biogenesis is crucial for the discrimination between pre-rRNAs that have to be processed and those of defective pre-ribosomal particles that have to be degraded [110]. If ribosome biogenesis proceeds, the lifetime of the intermediates is too short, to the extent that the surveillance machinery can attack the pre-rRNAs. However, if ribosome formation is blocked, stalled particles will accumulate activating the TRAMP complex for polyadenylation of the pre-rRNAs facilitating their degradation by the exosome.

Thus, inhibition of ribosome biogenesis by small molecular weight inhibitors not only impairs growth, but rapidly triggers the RNA surveillance system and mechanisms coping with unassembled r-proteins. Interestingly, in the human system, unassembled uL18 and uL5 bind to Mdm2, thereby preventing degradation of the tumor suppressor protein p53 [111]. The stabilization of p53 leads to cell cycle arrest and eventually apoptosis [111,112,113] (reviewed in [69,114]). Owing to the rapid manifestation of the drug effects, short treatment periods allow us to dissect the cellular responses step by step. This again highlights the necessity of investigating ribosome assembly in a high temporal resolution to understand the interplay of its regulatory circuits.

## 5. Concluding Remarks and Future Perspectives 

Eukaryotic ribosome biogenesis is one of the most intricate and dynamic processes within the cell with an enormously complex regulatory network behind it. Given the high interconnection between ribosome assembly and the cell cycle, chemical inhibition of this pathway provides the potential to target tumor cells due to their strict demand for a high supply of fresh ribosomes. Due to the stability of ribosomes, slowly proliferating cells have lower demands for freshly synthesized ribosomes and are therefore less sensitive to inhibition of ribosome biogenesis. Hence, targeting ribosome formation represents a promising strategy to selectively harm malignant cells. Besides being promising future anti-cancer drugs, ribosome biogenesis inhibitors bear the potential to bring the ribosome biogenesis research field forward in terms of development. Although we have a good idea of the basic principles, a complete and precisely drawn map unraveling the chronological order of maturation factor joining or coordination of individual steps is still desirable. While the development of cryo-EM analysis demonstrated the impressive movements and rearrangements occurring during maturation, they only provide static snapshots of distinct stages. This review aimed to highlight the power of small molecular weight compounds to investigate this complex process with a high temporal resolution which will enable us to combine the snapshots into a flipbook, going from static to dynamic. Although the currently available inhibitors were used for research in yeast so far, chemical probing apparently can also be used to investigate the even more complex ribosome biogenesis pathway in higher eukaryotes. 

Since diazaborine acts on the first stage in cytoplasmic maturation and hinders the recycling of shuttling factors, this inhibitor is highly suitable to investigate the communication between two sites of ribosome biogenesis, the cytoplasm and the nucleus. Rbin-1, the inhibitor of Rea1/Mdn1, conversely, will be extremely helpful for dissecting the individual tasks of this giant ATPase and will therefore probably provide deeper insights into the coordination of nucleoplasmic and nucleolar steps. In general, given the wealth of information already derived from the small number of specific inhibitors we have had at hand until recently, it is promising to look forward to expanding this knowledge by applying a much broader toolset. In this light, the characterization of the mode of action of the recently identified inhibitors of ribosome biogenesis [81] will have a major impact on the ribosome biogenesis research field and will pave the way for a detailed understanding of the dynamic nature of this impressive pathway.
